# Changes in Body Weight in Severely Obese Patients Treated with the Anorexiant Mazindol

**DOI:** 10.3390/jcm13071860

**Published:** 2024-03-23

**Authors:** Yoshimitsu Tanaka, Norikazu Maeda, Masahiro Koseki, Kazuhisa Maeda

**Affiliations:** 1 Longwood Maeda Clinic, Suita 565-0874, Japan; 2 Department of Endocrinology, Metabolism and Diabetes, Faculty of Medicine, Kindai University, Osaka-Sayama 589-8511, Japan; 3 Division of Cardiovascular Medicine, Department of Medicine, Osaka University Graduate School of Medicine, Suita 565-0871, Japan

**Keywords:** mazindol, obesity, anorexiant

## Abstract

(1) **Background**: The number of severely obese patients worldwide is rapidly increasing. Recently, novel therapeutic approaches, such as bariatric surgery or GLP-1 receptor agonists, have emerged, bringing about a paradigm shift in this field. However, these therapies sometimes face challenges, such as peri-surgical complications or supply shortages. Mazindol, which is an appetite suppressant approved decades ago in Japan, remains a valuable option. In this study, we investigated the effectiveness of mazindol in reducing body weight in 147 patients, and we examined the factors influencing said effectiveness. (2) **Methods**: The patients were divided into four groups based on the treatment cycles they underwent: 1 cycle, 2 cycles, 3–5 cycles, and over 6 cycles. We compared the changes in body weight before and after the treatment among these four groups. Additionally, we sought to identify the factors correlated to the effectiveness of mazindol. (3) **Results**: The change in body weight was more pronounced in the group which underwent 3–5 cycles compared to the groups which underwent 1 cycle and 2 cycles; this change was also more pronounced in the group which underwent over 6 cycles compared to those which underwent 1 cycle. Furthermore, we observed a significant correlation between the initial body weight and the extent of body weight change. (4) **Conclusions**: Mazindol demonstrated effectiveness in reducing the body weight of patients in a cycle-dependent manner.

## 1. Introduction

The global prevalence of obesity has reached alarming levels [1], necessitating effective strategies for weight reduction. Patients with severe obesity (BMI over 35) are at a higher risk of developing complications such as type 2 diabetes [2,3], hypertension [4], cardiovascular disease [5,6], and hypoventilation [7,8], among others. Therapies aimed at reducing body weight are crucial in preventing these illnesses. However, achieving a significant weight loss is often challenging [9]. Metabolic and bariatric surgeries have emerged as viable therapeutic options for severe obesity that has proved unresponsive to traditional diet and exercise interventions. A recent report from Kimura et al. has shown that metabolic and bariatric surgeries can change patients’ eating behaviors [10].

Despite its potential, the effectiveness of this surgical approach varies widely among patients, with some individuals experiencing minimal weight reduction or none at all [11]. Moreover, psychiatric complications—including the exacerbation of pre-existing mental illness, like depression—can occur in patients who undergo these surgical procedures [12,13,14]. 

In light of these challenges, anorexiant agents have long been recognized as a second-line treatment option, alongside metabolic and bariatric surgeries, according to the latest Japanese guidelines on the treatment of obesity [15]. Anorexiant agents offer the potential for safe and effective weight loss, the alleviation of obesity-related complications, and a reduction in polypharmacy [16]. However, the development of effective anorexiant agents has been hindered by insufficient efficacy and the emergence of severe side effects. While several anorexiant agents have gained approval from the U.S. Food and Drug Administration (FDA), mazindol has remained, for decades, the only approved anorexiant in Japan for highly obese patients, that is, until the recent approval of semaglutide. Despite its approval over 30 years ago, there is limited evidence regarding the clinical efficacy of mazindol worldwide. This is probably because its use is limited to a few countries, such as Japan, Canada, Mexico, and several countries in Middle and South America. Although two studies regarding the use of mazindol in Mexico have recently been published [17,18], there has been no report studying its efficacy in more than 100 cases in an Asian population. 

In this retrospective study, we aimed to investigate the effectiveness of mazindol by analyzing changes in body weight and the rate of body weight change. By retrospectively examining our data, we sought to contribute to the existing body of evidence on mazindol and to provide insights into the clinical efficacy of mazindol as an anorexiant agent for severely obese patients in Japan. 

## 2. Materials and Methods

Study design: This retrospective study aimed to evaluate the effectiveness of mazindol therapy in patients with severe obesity (i.e., BMI over 35). Patients who received mazindol treatment between August 2013 and April 2021 were enrolled consecutively. The inclusion criteria included a BMI over 35 and a minimum prescription term of 90 days. Patients with a history of metabolic and bariatric surgeries and those with insufficient clinical data were excluded from the analysis. Additionally, patients with a history of diseases contraindicated for mazindol, such as mental disorders including eating disorders, glaucoma, severe heart disease, severe pancreatic disease, severe kidney disease, severe liver diseases, drug and alcohol addiction, and severe hypertension, were excluded.

Mazindol therapy: Prior to initiating mazindol therapy, the patients’ baseline biochemical data were evaluated through blood samples. The patients were prescribed mazindol (FUJIFILM Toyama Chemical Co., Ltd., Tokyo, Japan) at a dosage of 0.5–1.5 mg once a day, to be taken before lunch. The prescription was repeated every two weeks for a total of 3 months, followed by a rest period of 3 months or more. The treatment was repeated depending on the patients’ preferences.

Weight assessment: The patients were categorized into four groups according to their number of treatment cycles, and the reduction in their body weight was assessed before and after they completed the treatment. The change in body weight (CBW) was calculated by subtracting their weight at the beginning from their weight at the end of each treatment cycle. The percentage change in body weight (%CBW) was calculated by determining the percentage of weight change relative to their baseline weight.

Data analysis: Descriptive statistics were used to summarize the quantitative variables, which are presented as mean values accompanied by standard errors. The categorical variables were summarized as percentages. Statistical significance was determined using a *p*-value threshold of less than 0.05. For the comparisons of background factors among groups, Fisher’s exact test was employed for nominal variables. Additionally, the Kruskal−Wallis test was used for continuous variables. For the pairwise comparison, the Bonferroni test was conducted. The correlation between the CBW (or %CBW) and the continuous variables was assessed using the Spearman’s rank correlation method. The relationship between the CBW (or %CBW) and the nominal variables, such as sex, comorbidities, and medications, was compared using the Mann−Whitney U test. The data analysis was conducted using the EZR software (version 1.61, Saitama Medical Center, Jichi Medical University, Saitama, Japan), which provides a user-friendly interface for statistical analysis. The software incorporates R (version 4.3.1, The R Foundation for Statistical Computing, Vienna, Austria) and the R commander (version 2.9-0, McMaster University, Hamilton, ON, Canada), allowing for the implementation of a wide range of statistical tests and procedures.

## 3. Results

### 3.1. Demographic Data

**Figure 1:**
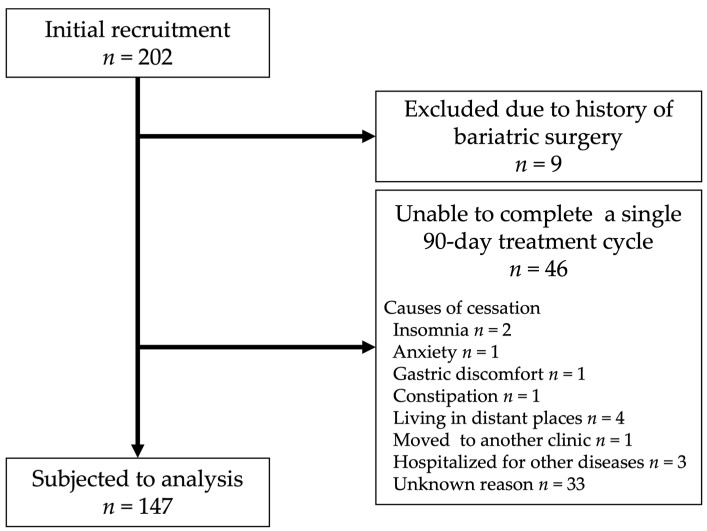
Enrollment of the study subjects. Initially, 202 patients were consecutively enrolled. Of these, 9 patients were excluded due to a history of bariatric surgery. A total of 46 patients were unable to complete a single 90-day treatment cycle (The causes of treatment cessation and number of cases are indicated in the same box). Finally, 147 patients were subjected to the retrospective analysis.

Of the total, 202 patients were consecutively enrolled. Among them, 9 were excluded due to a history of bariatric surgery and 46 dropped out before completing a single 90-day treatment cycle (2 due to insomnia, 1 due to anxiety, 1 due to gastric discomfort, 1 due to constipation, 4 due to living in a distant place, 1 moved to another clinic, 3 were hospitalized for other diseases, and 33 for unknown reasons) (Figure 1). As a result, a total of 147 patients were included in the retrospective analysis. The patients were categorized into four groups based on the number of treatment cycles they underwent (1 cycle, 2 cycles, 3–5 cycles, and over 6 cycles).

### 3.2. Baseline Characteristics

The number of patients in each group was as follows: *n* = 77 (1 cycle), *n* = 35 (2 cycles), *n* = 22 (3–5 cycles), and *n* = 13 (over 6 cycles) as indicated in Table 1. The BMI values were significantly higher in the over-six-cycle group compared to one-cycle and two-cycle groups. The prevalence of HL was significantly lower in the one-cycle group than the over-six-cycle group, and SAS was significantly lower in the one-cycle group compared to all other groups. The use of statins differed among groups, although there was no significant difference among groups upon pairwise comparison.

### 3.3. Change in Body Weight (CBW) and Percent Change in Body Weight (%CBW)

**Figure 2:**
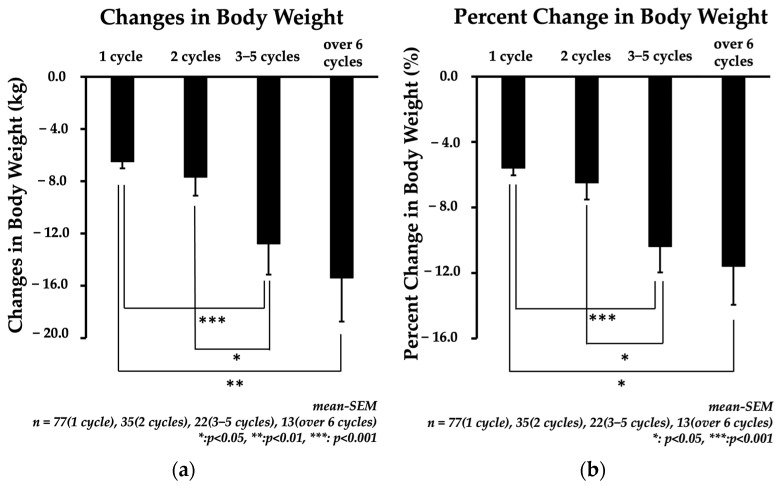
Effectiveness of mazindol therapy in each group divided by the number of treatment cycles performed. Graph (**a**) shows the changes in body weight (in kg), and graph (**b**) shows the proportions of the body weight changes (as percentages).

The changes in body weight observed in each treatment group were as follows: −6.5 ± 0.5 kg for 1 cycle; −7.7 ± 1.4 kg for 2 cycles; −12.8 ± 2.4 kg for 3–5 cycles; and −15.4 ± 3.3 kg for over 6 cycles (Figure 2a). The percent change in body weight observed in each treatment group was as follows: −5.6 ± 0.4% for 1 cycle; −6.5 ± 1.0% for 2 cycles; −10.4 ± 1.6% for 3–5 cycles; and −11.6 ± 2.3% for over 6 cycles (Figure 2b). In both CBW and %CBW values, we observed a significant increase in the effectiveness of the treatment in the over-six-cycle group compared to the one-cycle group. Additionally, we noted a significant increase in the 3–5-cycle group compared to the 1-cycle and 2-cycle groups. Overall, the effectiveness of the treatment seemed to increase as the patients went through multiple cycles.

### 3.4. Factors Influencing CBW and %CBW

**Figure 3:**
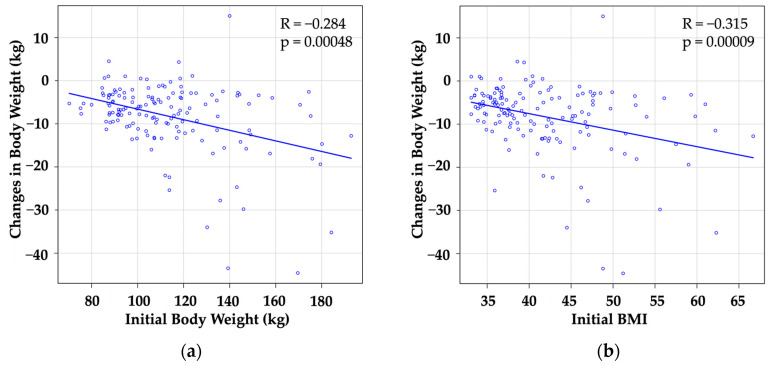

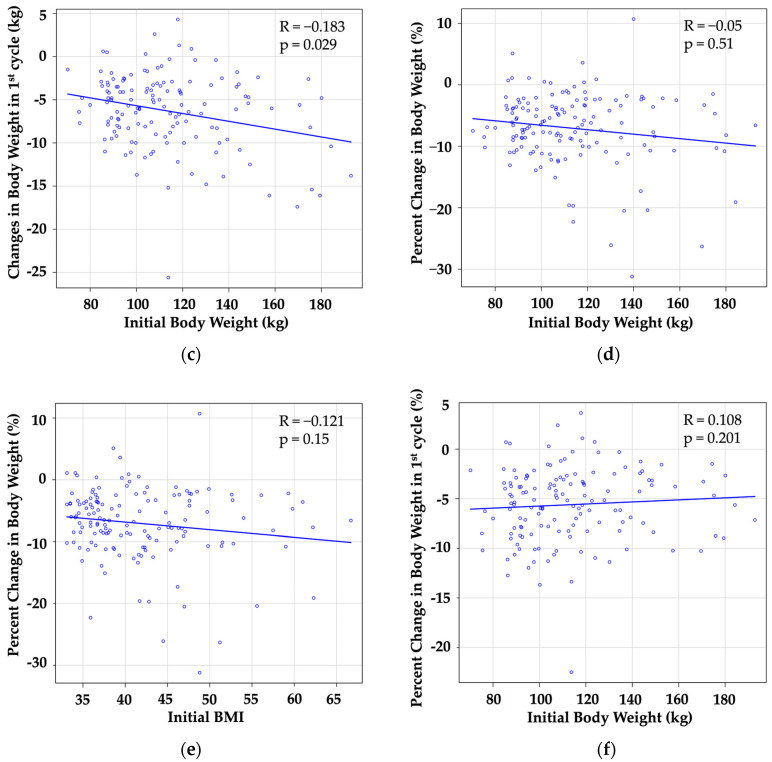
Relationship between the effect of mazindol, the initial body weight, and BMI.The upper two panels show the relationship between changes in body weight (CBWs) and initial body weight (**a**) or initial BMI (**b**). To exclude the effect of the number of treatment cycles, we analyzed the relationship between the initial body weight and CBWs only within the first cycle (**c**). In the latter three panels, the same analyses were performed, but only CBWs are replaced with percent change in body weight (**d**–**f**). (The blue circle corresponds to each patient.)

Next, we investigated the factors influencing the CBW. Among them, the initial body weight (Figure 3a) and initial BMI (Figure 3b) were found to be correlated with the CBW. As patients with higher body weight tended to undergo more treatment cycles, we analyzed the effect of mazindol within the first single cycle among 141 patients whose data were available. As a result, the initial body weight still correlated with the CBW after a single treatment cycle (Figure 3c). However, when we analyzed the effect of the treatment on %CBW, we did not find this correlation (Figure 3d–f).

Additionally, in patients with sleep apnea syndrome (SAS), the CBW was found to be more pronounced; meanwhile, other comorbidities, laboratory factors, and medications were not shown to affect the CBW (Figure S1). As the initial body weight may be a confounding factor for the CBW, we divided the CBW by the initial body weight (which is equal to the %CBW) and compared these values among patients with or without SAS; consequently, we found no significant difference between the two groups (Figure S2). (Data analyzing the relationships between the clinical factors and the CBW are shown in a supplementary file as Figure S1. Data analyzing the relationship between SAS and the CBW or %CBW are shown in Figure S2.)

## 4. Discussion

In this analysis of 147 cases of mazindol use in Japan, we examined the extent and rate of body weight changes. The results of this study highlight the significance of mazindol as a potential treatment option for severely obese patients. The data clearly demonstrate that the efficacy of mazindol in reducing body weight increases with the number of treatment cycles that the patient undergoes. While a significant correlation was observed between changes in body weight and the initial body weight, such a correlation was not found with the percentage change in body weight. As patients with a larger initial body weight tended to undergo more treatment cycles, we chose to analyze these correlations within a single treatment cycle. The results still show a significant correlation between changes in body weight and the initial body weight, but not in the percentage change in body weight. These findings suggest that patients who continue mazindol therapy over multiple cycles are more likely to achieve a substantial weight loss, maintaining a consistent ratio irrespective of the initial body weight. In light of said findings, mazindol may be a valuable therapeutic approach for individuals struggling with severe obesity.

Since mazindol has only been approved and used in limited regions, such as Japan, Canada, Brazil, and Mexico, among others, reports on the efficacy and safety of mazindol that feature a substantial number of cases remain rare. In 2022, reports from Mexico comparing the effects of mazindol with or without the concurrent use of metformin in 137 cases showed weight reductions of approximately 7–8% after 3 months of mazindol use and of 9–11% after 6 months of use [17]. Similarly, a 2022 report from Mexico involving 196 cases reported weight reductions of approximately 8.0% after 3 months of mazindol use and 10.7% after 6 months of use [18]. These reports indicate more pronounced reductions in weight when compared to the current study.

The differences in these effects may be influenced by several factors. In our study, most patients received a dosage of 0.5 mg of mazindol, while in previous studies in Mexico, 1.0 mg was administered in all cases. Additionally, the proportion of females in our study was 55%, while in the previous studies, it exceeded 80%. These differences, along with potential racial variations, may have contributed to the observed variations in weight reduction effects, although we did not find any gendered difference in body weight reduction.

It is important to note that the absolute body weight change exhibited a statistically significant negative correlation with the initial body weight and BMI. Furthermore, to distinguish the effect of duration, we analyzed the relationship between changes in weight before and after the first treatment cycle in all 141 cases whose data were available; therefore, we found that significant negative correlations were preserved. However, these correlations were lost when we used the percentage change in body weight as a marker of therapeutic effect. The results suggest that the body weight-reducing effect of mazindol is proportionally similar, irrespective of the pretreatment body weight.

When observing the overall results of this study, we can suggest that the therapeutic effect of mazindol leads to a consistent weight loss regardless of the initial body weight. Even in patients with a higher body weight, achieving their target body weight may be possible after multiple treatment cycles.

While previous reports have indicated that the weight reduction effects of mazindol are enhanced when the drug is used in combination with metformin [17], it is noteworthy that, in our study, there was no significant difference observed between the group receiving the metformin treatment and the group not receiving the metformin treatment. This may be because of the smaller number of diabetes or prediabetes patients treated with metformin in the current study. Further investigations exploring the potential impact of metformin in specific subpopulations or treatment regimens are therefore warranted.

Furthermore, our study found no significant impact on absolute weight loss associated with the use of GLP-1 receptor agonists or SGLT2 inhibitors as adjuvant therapies.

One limitation of our study was the very low number of patients using these medications. It is also possible that, by the time the mazindol treatment commenced, the weight-reducing effect of these medications had already reached a plateau. While GLP-1 receptors are expressed in multiple areas in the brain, such as the parietal cortex, hypothalamus, and brain medulla [19], mazindol’s sites of action are located in the hypothalamic lateral nucleus and the hypothalamic ventromedial nucleus [20,21], meaning a disparity in these drugs’ action sites. SGLT2 inhibitors act in the proximal tubules of the kidney [22]. Due to these differences in action sites, we anticipate a synergistic effect of mazindol when used in combination with these medications. Further investigations involving a larger number of cases are warranted in the future.

It is important to acknowledge that the influence of these medications on weight reduction outcomes may be affected by various factors, including the specific patient population, dosages, and treatment regimens. While our study did not identify a substantial effect on weight loss related to the use of these medications, further research may be necessary to explore their potential impact on specific patient subsets under different treatment conditions.

On the other hand, it was found that patients with SAS demonstrated greater reductions in weight. As the group with SAS had significantly larger initial body weight values, we divided the CBW by the initial body weight and compared the results among the two groups; thus, we found no significant difference. This suggests that the factor of initial body weight plays a more substantial role in the effects of mazindol.

Recently, semaglutide has been approved as an anti-obesity drug in Japan; it is the first drug of its kind to be authorized in the decades following the approval of mazindol. However, there do exist concerns about weight regain after the cessation of treatment. Combining mazindol with semaglutide may offer a solution for long-term weight management without significant rebounding effects.

Our study is unique in that it evaluated the effect of mazindol over an extended period of six cycles or more, contributing to its novelty. Since evidence of mazindol’s effect in Asian populations is limited, with the existing sample sizes ranging from 13 to 36 cases [23,24], the current study may contribute to establishing a new body of evidence for the effectiveness of mazindol in Asian populations.

In Japan, the administration of mazindol mandates bi-weekly clinic visits within a three-month drug cycle, followed by a rest period of over three months before the next cycle. This regimen prevents patients from cessation, helps to maintain motivation, and may contribute to long-term weight reduction effects.

### Limitations

This retrospective observational study was conducted within authentic clinical settings, lacking a randomized case–control design. Consequently, the absence of a placebo group poses challenges in discerning the genuine impact of the drug from other factors, such as the placebo effect, dietary restrictions, or exercise effects. A more refined evaluation of the effectiveness of mazindol necessitates a randomized case–control study.

## 5. Conclusions

In conclusion, this retrospective study demonstrates that mazindol may be an effective option for reducing body weight in severely obese patients. The treatment’s efficacy is positively correlated with the number of treatment cycles undertaken, highlighting the importance of continued therapy in the pursuit of optimal results. These findings contribute to our understanding of mazindol’s role in the management of severe obesity and emphasize the need for further research to explore its long-term effects and impact on obesity-related comorbidities.

## Figures and Tables

**Table 1:** Baseline characteristics of the subjects in this study.

Clinical Parameters	1 Cycle	2 Cycles	3–5 Cycles	Over 6 Cycles	*p*-Value	Pairwise Comparison
*n*	77	35	22	13	−	
Age (years)	44.1 ± 1.4	45.0 ± 2.1	44.8 ± 2.9	53.3 ± 3.4	0.14	n.s.
Sex (male/female)	41/36	23/12	13/9	4/9	0.55	n.s.
Height (cm)	164 ± 1.0	165 ± 1.7	165 ± 1.8	163 ± 2.1	0.86	n.s.
Weight (kg)	110 ± 2.6	111 ± 4.1	123 ± 6.6	124 ± 7.4	0.09	n.s.
BMI	40.8 ± 0.8	40.5 ± 0.9	44.5 ± 1.7	46.8 ± 2.3	0.004	1 cycle, 2 cycles < over 6 cycles *
BS (mg/dL)	125 ± 5.6	106 ± 4.1	117 ± 9.4	132 ± 16.6	0.22	n.s.
HbA1c (%)	6.6 ± 0.2	6.1 ± 0.1	6.0 ± 0.3	6.6 ± 0.3	0.10	n.s.
Systolic blood pressure (mmHg)	140 ± 2.0	135 ± 3.1	140 ± 3.9	140 ± 3.6	0.68	n.s.
Diastolic blood pressure (mmHg)	85 ± 1.4	83 ± 1.8	87 ± 2.3	86 ± 3.7	0.98	n.s.
LDL cholesterol (mg/dL)	120 ± 4.1	123 ± 4.7	128 ± 6.4	115 ± 3.9	0.65	n.s.
HDL cholesterol (mg/dL)	48 ± 1.2	50 ± 1.8	49 ± 1.9	48 ± 2.9	0.71	n.s.
Triglyceride (mg/dL)	178 ± 11	178 ± 20	146 ± 15	145 ± 19	0.33	n.s.
Uric acid (mg/dL)	5.9 ± 0.2	6.2 ± 0.2	5.6 ± 0.3	5.5 ± 0.3	0.20	n.s.
AST (IU/L)	32 ± 1.7	42 ± 4.9	27 ± 2.4	30 ± 3.4	0.16	n.s.
ALT (IU/L)	44 ± 3.4	60 ± 8.9	34 ± 4.6	36 ± 3.5	0.60	n.s.
Comorbidities						
Diabetes mellitus (%)	33(42.9)	13(37.1)	11(50.0)	9(69.2)	0.23	n.s.
Hypertension (%)	30(39.0)	16(45.7)	13(59.1)	8(61.5)	0.22	n.s.
Hyperlipidemia (%)	18(23.4)	19(54.3)	12(54.5)	11(84.6)	<0.001	1 cycle < 2 cycles, 3–5 cycles *, 1 cycle < over 6 cycles ***
Sleep apnea syndrome (%)	10(13.0)	17(48.6)	13(59.1)	9(69.2)	<0.001	1 cycle < 2 cycles, 3–5 cycles, over 6 cycles ***
Medications						
Biguanide (%)	16(20.8)	8(22.9)	6(27.3)	6(46.2)	0.26	n.s.
SGLT2 inhibitor (%)	16(20.8)	7(20.0)	2(9.1)	5(38.5)	0.25	n.s.
DPP4 inhibitor (%)	7(9.1)	3(8.6)	2(9.1)	3(23.1)	0.45	n.s.
GLP1 receptor agonist (%)	5(6.5)	4(11.4)	3(13.6)	2(15.4)	0.45	n.s.
SU (%)	6(7.8)	1(2.9)	0(0)	1(7.7)	0.44	n.s.
Insulin (%)	6(7.8)	0(0)	0(0)	2(15.4)	0.06	n.s.
Angiotensin receptor blocker (%)	24(31.2)	9(25.7)	7(27.3)	8(61.5)	0.14	n.s.
Calcium channel blocker (%)	22(28.6)	5(14.3)	6(27.3)	6(46.2)	0.13	n.s.
Statin (%)	13(16.9)	5(14.3)	9(40.9)	6(46.2)	0.01	n.s.

Data are expressed as mean ± SE, numbers, or as frequencies (%). *: *p* < 0.05, ***: *p* > 0.001. n.s.: not significant

## Data Availability

The raw data supporting the conclusions of this article will be made available by the authors upon request.

## Supplementary Materials

The following supporting information can be downloaded at: https://www.mdpi.com/article/10.3390/jcm13071860/s1. Figure S1: Relationship between the CBW and comorbidities, laboratory factors, and medications. Figure S2: Effect of SAS on the CBW and %CBW.
