# Supplementary material for: Changes in Body Weight in Severely Obese Patients Treated with the Anorexiant Mazindol
Source: J Clin Med. 2024 Mar 23;13(7):1860. doi: 10.3390/jcm13071860 (PMC11012520; doi:10.3390/jcm13071860)
Supplement: Supplementary file 1 [file jcm-13-01860-s001.zip › jcm-2924026-supplementary.pdf]

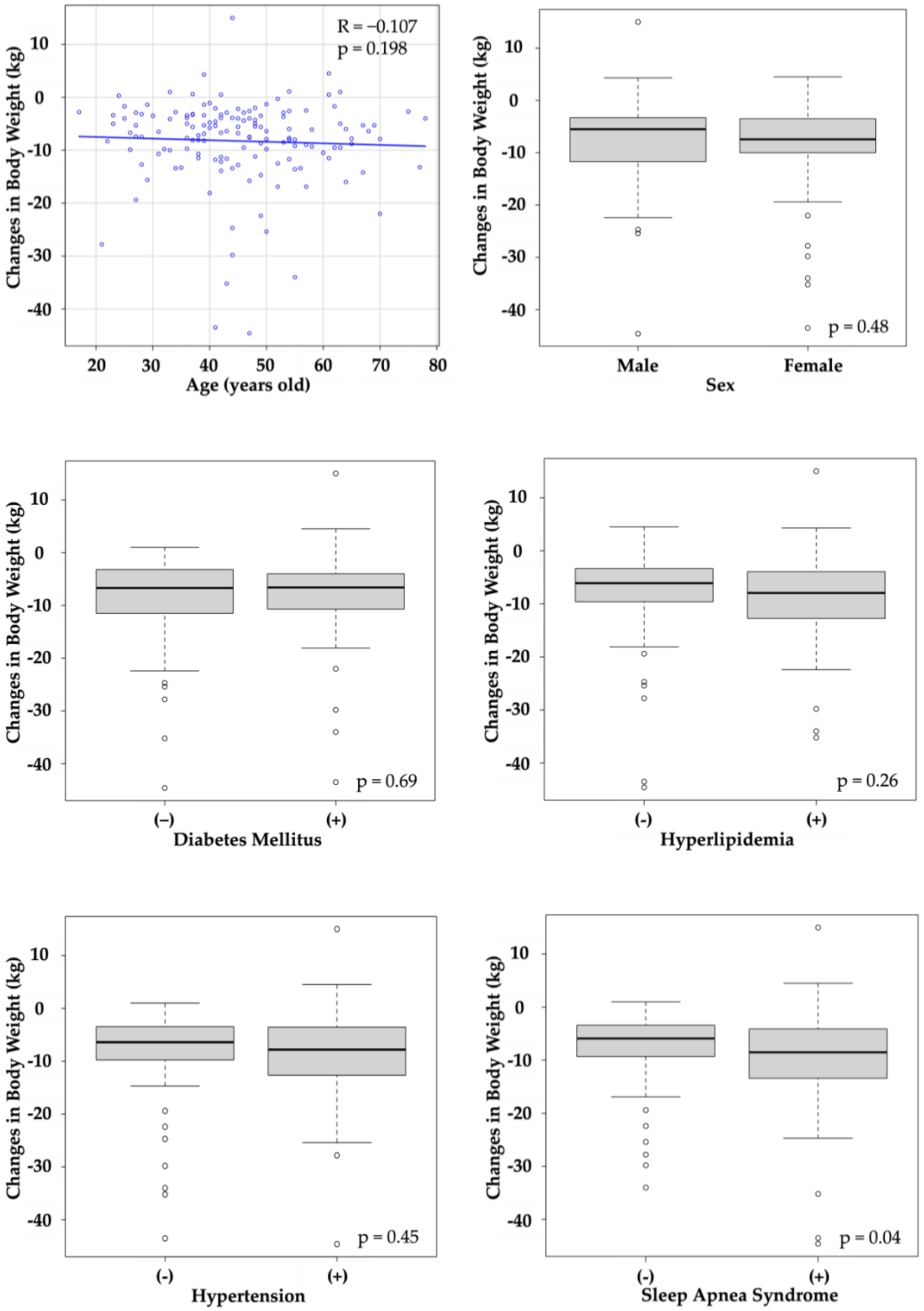
R = −0.107
p = 0.198
Changes in Body Weight (kg)
Age (years old)
p = 0.48
Male
Female
Sex
p = 0.69
(–)
(+)
Diabetes Mellitus
p = 0.26
Hyperlipidemia
p = 0.45
Hypertension
p = 0.04
Sleep Apnea Syndrome

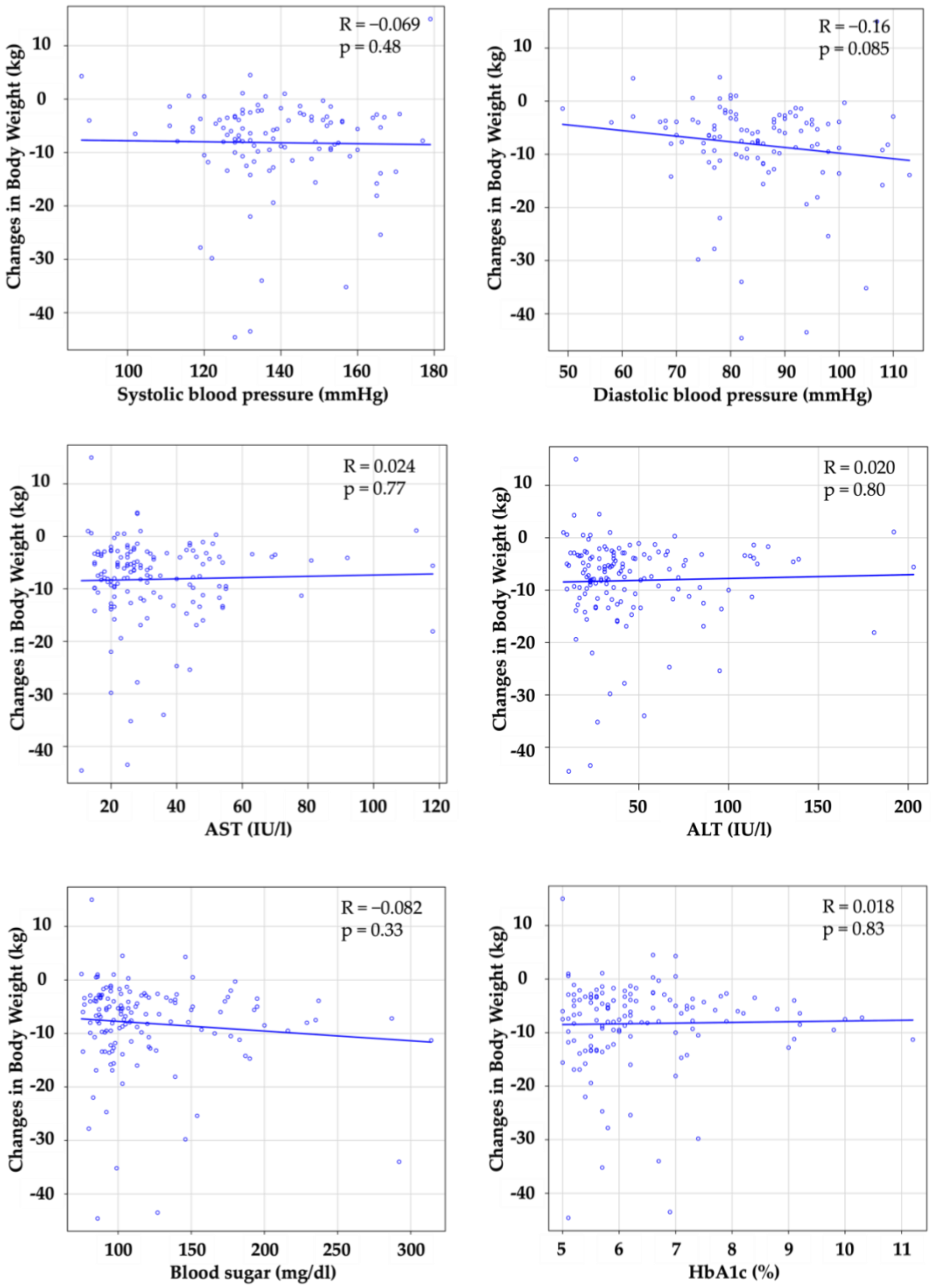
R = −0.069
p = 0.48
Changes in Body Weight (kg)
Systolic blood pressure (mmHg)
R = −0.16
p = 0.085
Changes in Body Weight (kg)
Diastolic blood pressure (mmHg)
R = 0.024
p = 0.77
Changes in Body Weight (kg)
AST (IU/l)
R = 0.020
p = 0.80
Changes in Body Weight (kg)
ALT (IU/l)
R = −0.082
p = 0.33
Changes in Body Weight (kg)
Blood sugar (mg/dl)
R = 0.018
p = 0.83
Changes in Body Weight (kg)
HbA1c (%)

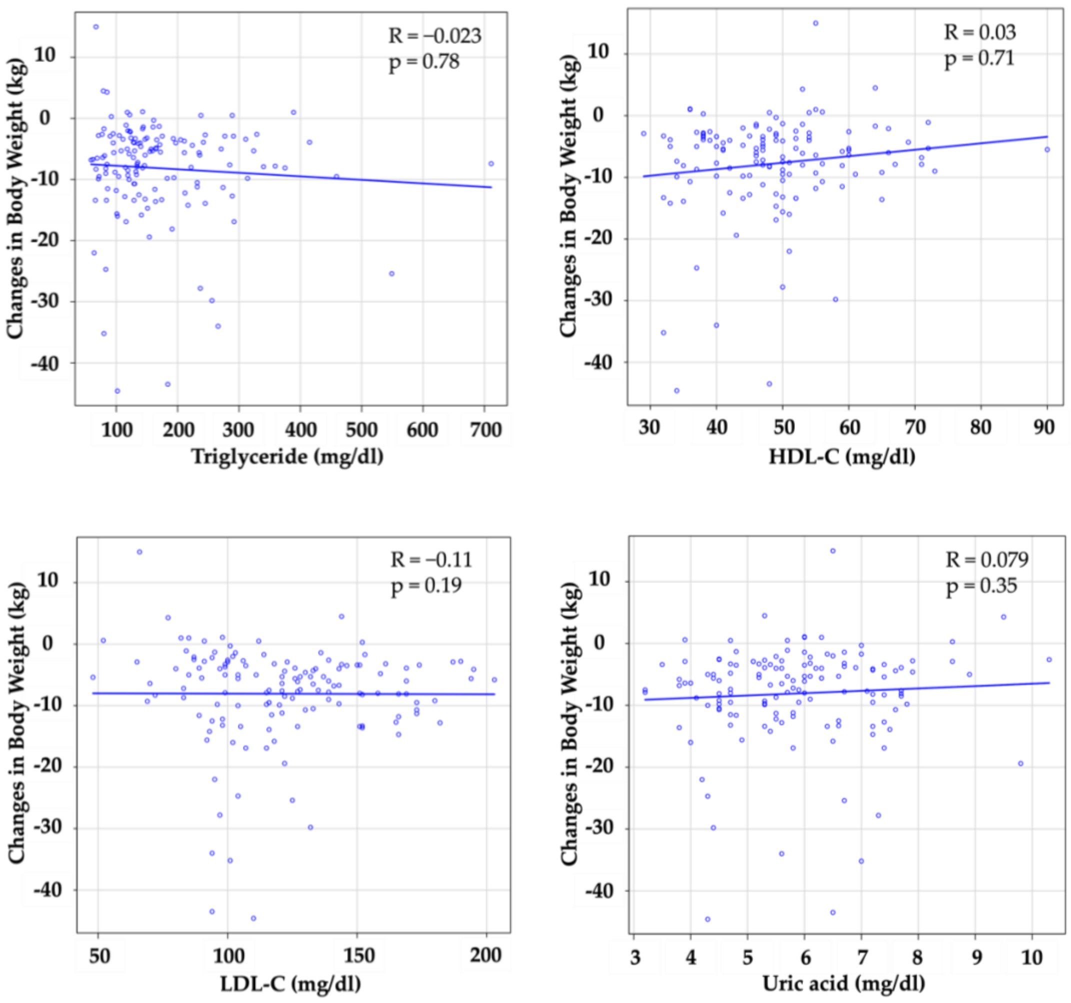

Figure S1. Relationships between the changes in body weight and comorbidities, laboratory factors, and medications.

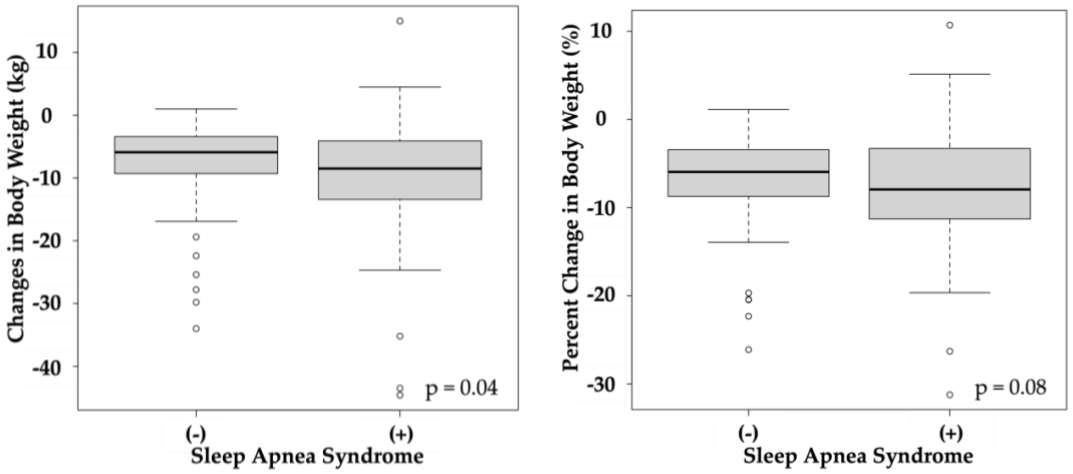

Figure S2. Effect of Sleep apnea syndrome on the changes in body weight (the left panel) and the proportions of the body weight changes (the right panel).
